# 

**Published:** 1849-01-01

**Authors:** 


### THE JOURNAL

OF

### PSYCHOLOGICAL MEDICINE

AND

### MENTAL PATHOLOGY.

EDITED BY

FORBES WINSLOW, M.D.

VOL. II.

Surgeon Genl's Office
LIBRARY.
22046
Washington, D.C.

LONDON:

JOHN CHURCHILL, PRINCES STREET, SOHO.

MDCCCXLIX.

W
1
J0 857 N

### LITERARY NOTICE.

We are pleased to announce that Dr. Calvert Holland, a gentleman well known to the profession, is preparing for publication a work entitled, "Practical Views on Nervous Affections, on the Principles of Physiology applied to the elucidation of Disease."

---

### BOOKS RECEIVED.

A History, Description, and Statistics, of the Bloomingdale Asylum for the Insane. By Pliny Earle, M.D., New York, 1848. (This work will be noticed at length in our next number.)
Annales Medico-Psychologiques. Paris, 1848.
Reports of the Bloomingdale Asylum.
State of the Lincoln Lunatic Asylum, 1848.
American Journal of Insanity — various numbers. (These will be reviewed in the next number.)
On the Study of Medicine, and the Duties of the Student.
An Introductory Lecture delivered at St. Bartholomew's Hospital. By William Baly, M.D., F.R.S.
Reports of the Trustees, Stewards, and Treasurer, and Superintendent, of the Insane Hospital, Augusta.
Report (Twenty-fourth) of the Trustees of the Maine Insane Hospital.
Introductory Lecture delivered at the Calcutta Medical College, 1848. By H. H. Goodeve, M.D., Fellow Roy. Coll. Surg. England, &c. (An interesting and able production, calculated to do much good, and advance the reputation of the author.)
The Dublin Quarterly Journal of Medical Science.
The Provincial Medical and Surgical Journal—regularly.
The Dublin Medical Press—regularly.
On the Use and Abuse of Restraint in the Management of the Insane. By H. Labatt, A.B., T.C.D. (Will be reviewed in our next.)
Treatment of Asiatic Cholera. By A. Billing, F.R.S. Second Edition.
A Critical Treatise on the General Paralysis of the Insane. By J. M. Winn, M.D., Physician to the Royal Cornwall Infirmary, and Truro Dispensary. (A Reprint from the Journal.)

### DEATH OF DR. J. C. PRICHARD.

It is our painful duty to record the death of Dr. Prichard, the distinguished Ethnologist, Psychologist, and Commissioner in Lunacy. His loss will be seriously felt and deeply deplored. We can ill afford to lose such a man. Dr. Prichard's principal works on "Insanity," and on the "Physical and Natural History of Man," have given him a European reputation. He was distinguished by all the characteristics which usually accompany greatness of mind. He was modest and unassuming, manifesting extreme kindness of heart and benevolence of disposition in all his official and social relations.

